# Aucubin alleviates oxidative stress and inflammation via Nrf2-mediated signaling activity in experimental traumatic brain injury

**DOI:** 10.1186/s12974-020-01863-9

**Published:** 2020-06-15

**Authors:** Han Wang, Xiao-Ming Zhou, Ling-Yun Wu, Guang-Jie Liu, Wei-Dong Xu, Xiang-Sheng Zhang, Yong-Yue Gao, Tao Tao, Yan Zhou, Yue Lu, Juan Wang, Chu-Lei Deng, Zong Zhuang, Chun-Hua Hang, Wei Li

**Affiliations:** 1 Department of Neurosurgery, Nanjing Drum Tower Hospital, The Affiliated Hospital of Nanjing University Medical School, 321# Zhongshan Road, Nanjing, 210008 China; 2 Department of Neurosurgery, Jinling Hospital, School of Medicine, Nanjing University, Nanjing, China; 3 Department of Neurosurgery, Jinling Hospital, School of Medicine, Nanjing University, Nanjing, China

**Keywords:** Traumatic brain injury (TBI), Aubucin, Oxidative stress, Inflammation, Nuclear factor erythroid-2 related factor 2 (Nrf2)

## Abstract

**Background:**

Aucubin (Au), an iridoid glycoside from natural plants, has antioxidative and anti-inflammatory bioactivities; however, its effects on a traumatic brain injury (TBI) model remain unknown. We explored the potential role of Au in an H_2_O_2_-induced oxidant damage in primary cortical neurons and weight-drop induced-TBI in a mouse model.

**Methods:**

In vitro experiments, the various concentrations of Au (50 μg/ml, 100 μg/ml, or 200 μg/ml) were added in culture medium at 0 h and 6 h after neurons stimulated by H_2_O_2_ (100 μM). After exposed for 12 h, neurons were collected for western blot (WB), immunofluorescence, and M29,79-dichlorodihydrofluorescein diacetate (DCFH-DA) staining. In vivo experiments, Au (20 mg/kg or 40 mg/kg) was administrated intraperitoneally at 30 min, 12 h, 24 h, and 48 h after modeling. Brain water content, neurological deficits, and cognitive functions were measured at specific time, respectively. Cortical tissue around focal trauma was collected for WB, TdT-mediated dUTP Nick-End Labeling (TUNEL) staining, Nissl staining, quantitative real time polymerase chain reaction (q-PCR), immunofluorescence/immunohistochemistry, and enzyme linked immunosorbent assay (ELISA) at 72 h after TBI. RNA interference experiments were performed to determine the effects of nuclear factor erythroid-2 related factor 2 (Nrf2) on TBI mice with Au (40 mg/kg) treatment. Mice were intracerebroventricularly administrated with lentivirus at 72 h before TBI establishment. The cortex was obtained at 72 h after TBI and used for WB and q-PCR.

**Results:**

Au enhanced the translocation of Nrf2 into the nucleus, activated antioxidant enzymes, suppressed excessive generation of reactive oxygen species (ROS), and reduced cell apoptosis both in vitro and vivo experiments. In the mice model of TBI, Au markedly attenuated brain edema, histological damages, and improved neurological and cognitive deficits. Au significantly suppressed high mobility group box 1 (HMGB1)-mediated aseptic inflammation. Nrf2 knockdown in TBI mice blunted the antioxidant and anti-inflammatory neuroprotective effects of the Au.

**Conclusions:**

Taken together, our data suggest that Au provides a neuroprotective effect in TBI mice model by inhibiting oxidative stress and inflammatory responses; the mechanisms involve triggering Nrf2-induced antioxidant system.

## Introduction

Traumatic brain injury (TBI) is a major public health problem in modern society, causing functional impairments and imposing heavy economic burdens to patients. The pathophysiology of TBI consists of primary and secondary brain injuries [1, 2]. Trauma-induced primary brain injury results in tissue damage and neuronal loss. Secondary brain injury occurs hours or days following primary brain injury and involves glutamate excitotoxicity, neuroinflammation, calcium overload, oxidative stress, and cell death [3]. Of these, oxidative stress (OS) and inflammatory reaction are recognized as the important pathobiological features of secondary brain damage [4, 5]. Excessive production of free radicals leads to lipid peroxidation, protein degradation, and genotoxicity leading to cellular and tissue damage [6]. The brain is an organ with a high content of polyunsaturated fatty acids, which also makes it vulnerable to free radical attack and lipid peroxidation [7]. In addition, OS damage to mitochondrial function can collapse the cellular bioenergetics leading to cell apoptosis [6]. These OS-induced damaged cells release damage associated molecular patterns (DAMPs; e.g., ATP, RNA, high-mobility group box 1) to initiate or exacerbate neuroinflammation, which can also further promote ROS generation [8, 9]. Extensive animal data suggest that elevated antioxidant response and reduced inflammation would attenuate brain damage [10–12].

Nuclear factor erythroid-2 related factor 2 (Nrf2) has considerable neuroprotective effects in central nervous system (CNS) diseases [13–15]. Nrf2 is a transcription factor that takes part in regulation of cellular response to oxidative stress. Under normal physiological conditions, Kelch-like ECH-associated protein 1 (Keap1) combines with Nrf2 and enhances Nrf2 degradation. Once stimulated, Nrf2 translocates into the nucleus and binds to the antioxidant response element (ARE) in the promoter of antioxidant genes, thereby inducing the expression of antioxidant and detoxification enzymes and downstream proteins such as heme oxygenase-1 (HO-1), NAD(P)H: quinone oxidoreductase-1 (NQO-1), glutathione peroxidase (GPx), glutathione-S-transferase (GST), and superoxide dismutase (SOD) [16–18].

High-mobility group box 1 (HMGB1) is a member of DAMPs, which instigates and amplifies the noninfectious inflammatory response following TBI [19]. After secretion by inflammatory cells and/or expulsion by damaged neurons into the extracellular milieu [20, 21], HMGB1 interacts with toll-like receptors (TLRs) on the microglia to initiate an immune response that actives myeloid differentiation factor 88 (MyD88) leading to translocation of nuclear factor-κB (NF-κB) dimers to the nucleus, where it binds to specific DNA sequences and promotes the transcription of inflammatory cytokine gene [22–24].

Aubucin (1,4a,5,7a-tetrahydro-5-hydroxy-7-hydroxymethylcyclopenta(c)pyran-1-yl-beta-D-glucopyranoside, Au) is a member of the iridoid glycoside family and is widely found in plant species. Several lines of evidence suggest that Au has a wide range of pharmacological properties including antioxidation, anti-aging, anti-inflammation, anti-fibrosis, anti-tumor, and hepatoprotection [25]. Recently, its anti-inflammatory and antioxidative functions have become a focus of research. Studies have shown that Au provides neuroprotective effects via anti-inflammation and antioxidative properties [26–30]. Nevertheless, its molecular mechanisms are not well understood. Recent reports have found that Au both exerts antioxidant and anti-inflammation properties by activating the Nrf2 signaling pathway in non-central nervous system diseases [31, 32]. However, to date, it remains unknown as to whether Au has the same protective effects on TBI. Therefore, we investigated the effects of Au on the secondary brain injury in an experimental mouse TBI model and tried to explore potential molecular mechanisms.

## Materials and methods

### Animals

Pregnant C57BL/6 mice at 16–18 days gestation and adult male C57BL/6 mice were purchased from the Animal Center of Drum Tower Hospital, Nanjing, China. All adult male C57BL/6 mice (25–30 g) were raised in a 12-h light/dark cycle environment with free access to food and water.

### Experiment design

#### Experiment 1

Seven pregnant mice and 65 fetuses were used in vitro experiments. The primary cortical neurons were randomly assigned into five groups: Control group, H_2_O_2_ group, H_2_O_2_ + Au groups (50 μg/ml, 100 μg/ml, 200 μg/ml) for protein extraction (*n* = 3 for each group). In addition, three groups of neurons: Control group, H_2_O_2_ group, and H_2_O_2_ + group (200 μg/ml) were used for immunofluorescence staining and M29,79-dichlorodihydrofluorescein diacetate (DCFH-DA) staining (*n* = 3 for each group).

#### Experiment 2

One hundred twenty mice (130 mice underwent the operation, 120 survived) were used in the experiments. The mice were randomly allocated into four groups: Sham group, TBI group, TBI + Au group (20 mg/kg and 40 mg/kg). We performed modified Neurological Severity Scores (mNSS), rotarod test, and Morris Water Maze (MWM) test, respectively (*n* = 8 for each group). The other mice were sacrificed 3 days after trauma to measure brain water content (BWC) (*n* = 6 for each group).

#### Experiment 3

Ninety-six mice (107 mice underwent the operation, 96 survived) were randomly assigned to four groups: Sham group, TBI group, TBI + Au group (20 mg/kg and 40 mg/kg). All mice in this experiment were sacrificed at 3 days after trauma. Six mice in each group were used for western blot (WB), quantitative real time polymerase chain reaction (q-PCR), and enzyme linked immunosorbent assay (ELISA), respectively (*n* = 6 for each group). Brain tissues from the remaining mice were used to make paraffin slices for TdT-mediated dUTP Nick-End Labeling (TUNEL) staining, Nissl staining, immunofluorescence (IF) staining, and immunohistochemistry (IHC) staining (*n* = 6 for each group).

#### Experiment 4

First, 48 mice (56 mice underwent the operation, 48 survived) were divided into four groups (*n* = 6, each group): Sham group, TBI group, TBI + negative control (NC) group, and TBI + lentiviral vectors (LV) group and sacrificed at 3 days after TBI for WB and q-PCR to verify the effectiveness of the Nrf2-specific shRNA. Then, 30 mice (35 mice underwent the operation, 30 survived) were randomly assigned to five groups (*n* = 6, each group): Sham group, TBI group, TBI + Au (40 mg/kg) group, TBI + NC + Au (40 mg/kg) group, and TBI + Lv + Au (40 mg/kg) group for WB.

### Primary cortical neuron culture

The skull, blood, and meninges were carefully removed from the fetal mice brain. After cortical tissue digested within 0.25% trypsin (Gibco, USA) for 5 min at 37 °C, the suspensions with fetal bovine serum (biological industries, USA) were passed through filters with 22 μm mesh size (Millipore, USA) and then were centrifuged at 1500 rpm for 5 min. The supernatant was discarded, and the pellets were resuspended in Dulbecco’s Modified Eagle Medium (Gibco, USA). The cells were distributed in poly-D-lysine-coated plates. Four hours later, the medium was replaced with neurobasal medium supplemented with streptomycin, penicillin, HEPES, glutamate, and B27 (Gibco, USA). Half of the neurobasal medium was refreshed every 2 days. After 7–8 days culture, neurons were used in vitro experiments.

### In vitro and in vivo model establishment

For in vitro studies, the primary cortical neurons were incubated with H_2_O_2_ dissolved in neuronal culture medium at a final concentration of 100 μM for 12 h according to published research with minor modification [33]. Then, the neurons were collected for WB, IF staining, and DCFH-DA staining. For in vivo experiments, we used the TBI model induced by weight drop. Mice were anesthetized with intraperitoneal sodium pentobarbital (40 mg/kg) and then placed onto the platform of the weight-drop apparatus. After disinfection, mice scalps were cut with a longitudinal midline incision to expose the skull. Then, the weight-drop device with a 200 g was released from a height of 2.5 cm, to cause focal trauma on the hemisphere 1.5 mm lateral to the midline on the mid-coronal plane. Mice with scalp incisions were sutured and returned to their cages and awaked from anesthesia. Mice in Sham group underwent the same procedures except for the weight drop.

### Drug administration

For in vitro studies, Au (cat# HY-N0664, MedChemExpress, USA) was dissolved in neuronal culture medium at concentrations of 50 μg/ml, 100 μg/ml, or 200 μg/ml. After stimulating cells with 100 μM H_2_O_2_, the various concentrations of Au were added immediately and given again at 6 h after H_2_O_2_ administration. For in vivo studies, Au was dissolved in normal saline to reach final concentrations of 4 mg/ml. The intraperitoneal injection of Au (20 mg/kg or 40 mg/kg) was at 30 min, 12 h, 24 h, and 48 h after TBI. In the RNA interference experiments, the mice were intraperitoneally injected with Au (40 mg/kg) at 30 min, 12 h, 24 h, and 48 h after TBI establishment.

### Preparation of paraffin-embedded sections

Anesthetized mice were perfused 0.85% solution followed by 4% paraformaldehyde, and the brain tissues were removed. After immersion in 4% paraformaldehyde for 24 h, the brain tissues were dehydration in 45%, 55%, 65%, 75%, 85%, 95%, and 100% ethanol for 30 min, respectively. Then, the tissues were vitrification in 50% xylene (diluted with 100% ethanol) for 10 min and 100% xylene twice for 40 min. Then, brain tissues were made into paraffin blocks that were cut into 6 μm sections. The paraffin sections were dewaxed in 100% xylene twice for 10 min, 100% ethanol twice for 10 min, 95% ethanol for 5 min, 85% ethanol for 5 min, and 70% ethanol for 5 min, respectively. After washing two times with distilled water for 10 min, the slices were boiled in a microwave with citrate buffer solution (cat# P0083, Beyotime, China) for 15 min to retrieve antigens and then were used for IF staining, Nissl staining, TUNEL staining, and IHC staining.

### WB analysis

The neurons and brain tissue (Additional file 1: Figure S1) were collected for WB analysis. The procedure of nuclear and total protein extraction was according to manufacturer’s instructions. Equal protein amounts were separated using polyacrylamide gel electrophoresis and transferred to polyvinylidene difluoride membranes blocked in 5% skim milk for 2 h at indoor temperature. Then, the membranes were incubated with primary antibodies against Nrf2 (1:1000, cat# ab137550, Abcam), Histone H3 (H3) (1:5000, cat# AF0009, Beyotime, China), NQO-1 (1:1000, cat# ab34173, Abcam), HO-1 (1:1000, cat# ab13248, Abcam), B-cell lymphoma-2 (Bcl2) (1:200, cat# 196495, Abcam), Bcl-2 Associated X Protein (Bax) (1:200, cat# ab32503, Abcam), cleaved-caspase 3 (CC3) (1:1000, cat# 9664, Cell Signaling), matrix metalloprotein-9 (MMP-9) (1:1000, cat# PB10008, Boster), glutathione peroxidase 1 (GPx1) (1:1000, cat# ab22604, Abcam), superoxide dismutase 1 (SOD1) (1:200, cat# sc-101523, Santa Cruz), Iba-1 (1:1000, cat# ab178846, Abcam), HMGB1 (1:100, cat# sc-56698, Santa Cruz), TLR4 (1:200, cat# sc-30002, Santa Cruz), MyD88 (1:200, cat# sc-74532, Santa Cruz), NF-κB p65 (1:1000, cat# 8242, Cell Signaling), inducible Nitric Oxide Synthase (iNOS) (1:1000, cat# 13120, Cell Signaling), Cyclooxygenase-2 (COX2) (1:500, cat# sc-19999, Santa Cruz), Interleukin-1β (IL-1β) (1:500, cat# 12242, Cell Signaling), or β-actin (1:3000, cat# BS6007M, Bioword) overnight at 4 °C. After washing 3 times for 15 min with Tris-buffered saline with Tween 20, the membranes were incubated with corresponding HRP conjugated secondary antibodies (1:5000, Bioword) for 1 h at room temperature. The protein bands were detected using enhanced chemiluminescence (ECL). The ImageJ software was used to measure the optical density of protein bands.

### IF staining

The brain sections with antigen retrieval or cultured cells were treated with QuickBlock™ Blocking Buffer (cat# P0260, Beyotime, China) for 30 min at room temperature and then incubated with primary antibodies against Nrf2 (1:100, cat# ab137550, Abcam), MAP2 (1:200, cat# sc-32791, Santa Cruz), NeuN (1:200, cat# MAB377, EMD Millipore), and Iba-1 (1:100, cat# ab178846, Abcam) overnight at 4 °C. The cells or slides were incubated with corresponding secondary antibodies Alexa Fluor 594 and/or Alexa Fluor 488 (1:200, Jackson ImmunoResearch Incorporation, West Grove, PA, USA). Then, the cells or slides were washed in phosphate buffered saline-Tween twice for 20 min and counterstained with 4,6-diamidino-2-phenylindole (DAPI) (1:2000, cat# ab104139, Abcam) for 5 min. Fluorescence was captured on a Zeiss HB050 inverted microscope system. The ImageJ software was used to measure the inflorescence intensity.

### DCFH-DA staining

The procedures of ROS measurement were according to manufacturer’s instructions. After stimulating with H_2_O_2_ for 12 h, the neurons were incubated with DCFH-DA (cat# S0033, Beyotime, China) for 10 min at 37 °C. Following three washes with serum-free medium (Gibco, USA) for 1 min, the cells were immediately photographed under an inverted fluorescence microscope. The mean fluorescence intensity was analyzed using the ImageJ software.

### IHC staining

After antigens retrieval, the sections were treated with 3% H_2_O_2_ for 15 min and QuickBlock™ Blocking Buffer (cat# P0260, Beyotime, China) for 30 min at room temperature. Then, sections were incubated with primary antibodies against HO-1 (1:200 cat# ab13248, Abcam), NQO1 (1:200, cat# ab34173, Abcam), and 8-hydroxyguanosine (8-OHdG) (1:200, cat# sc-393871, Santa Cruz) overnight at 4 °C. The slides were washed twice with phosphate-buffered saline (PBS) and incubated with biotinylated secondary antibody (1:500, Vector, Burlingame, CA, USA) and horseradish peroxidase (HRP)-streptavidin reagent (Vector, USA). Then, the sections were re-stained with hematoxylin, and we measured immunoreactivity using 3,3-diaminobenzidine (DAB, Zsgb-bio, China). Images were pictured by a microscope. The ImageJ software was used to analyze the IHC images.

### Brain water content

The brain water content was performed at 3 days after TBI. The entire brain was divided into injured hemispheres, cerebellum, and brainstem. Each part was weighed immediately to obtain the wet weight. Brain tissue was dried at 80 °C for 72 h and re-weighed again to calculate dry weight. The brain water content was calculated as [(wet weight − dry weight)/wet weight] × 100%.

### Neurologic function testing

The mice were assessed using mNSS test on days 1 and 3 after TBI according to previous studies [34]. The mNSS consists of motor (muscle status and abnormal movement), sensory (visual, tactile, and proprioceptive), reflex, and balance tests. One score is awarded for the inability to perform the test or for the lack of a tested reflex. The higher score represents the more serious neurological impairment (normal score, 0; maximal score, 18). Detailed score criteria was shown in Additional file 1: Figure S2.

### MWM test

On the 24th day after the trauma, the mice were performed the MWM test with minor modifications to assess their cognitive functions. Visual cues of figures were hung on the wall of the tank. During the acquisition phase, the mice were continuously trained for 5 days, with 3 trials per day. In each trial, mice were given 1 min to find a submerged platform 1 cm below the surface of the water. When arrival to the platform, mice were allowed to stay on the platform for 15 s. If failed, mice were guided to the platform and stay for 30 s. The mice were subjected to the probe trials on next day after 5 consecutive training. The platform was removed from the tank, and then, mice were placed in the quadrant opposite the platform to seek platform for 1 min. The ANY-Maze video tracking system was used to videotape the whole process and data.

### Rotarod test

Mice were received 3 days of rotarod test training before TBI induction and then placed in a neutral position on an accelerating rotating rod (from 5 to 40 r/min within 5 min). The blinded experimenters recorded the latency to fall of each mouse. An average latency of three trials in 1 day represented the mouse motor performance. The test was performed before TBI and 0, 3, 7, and 14 days following TBI.

### TUNEL staining and Nissl staining

Antigen-repaired brain sections were incubated with TUNEL reaction mixture (cat# C1089, Beyotime, China) for 1 h at room temperature. After two washes with PBS for 20 min, the slides were incubated with DAPI for 5 min. Then, the brain sections were washed twice again and exposed under an inverted fluorescence microscope. The TUNEL-positive cells were counted by the ImageJ software. The number of apoptosis cells to DAPI was regarded as an apoptosis index (apoptosis cells/DAPI). For Nissl staining, the sections were stained in Nissl staining solution (cat# C0117, Beyotime, China) for 5 min, washed with double-distilled water, and mounted with Permount. The pictures were captured under a light microscope. The neurons with visible nuclear and relatively complete cellular morphology were counted by the ImageJ software.

### Contents of malondialdehyde (MDA), SOD, ROS, GSH, and GSH-Px measurements

Levels of MDA, SOD, ROS, GSH, and GSH-Px in serum and brain tissue were measured using ELISA kits (Nanjing Jiancheng Bioengineering Institute, Nanjing, China) according to the manufacturer’s instructions at 3 days after TBI.

### RNA interference

The transfection of LV expressing Nrf2-specific shRNA or negative control shRNA for mice in vivo was conducted 3 days prior to TBI modeling. After intraperitoneal anesthesia with sodium pentobarbital (40 mg/kg), mice with scalp incisions were placed in a stereotaxic device; then, a cranial burr hole (2.5 mm in depth, 1.2 mm lateral from midline, and 0.4 mm posterior from the bregma) was drilled. The mice underwent injection with 4 μL lentiviruses into the lateral ventricles (2 μL/min). The needle remained in place for 30 s after completing the infusion, and the scalp incision was sutured. The mice in Sham, TBI, and TBI + Au groups received a cranial burr hole but not intracerebroventricular (ICV) injection. The experimental TBI was established 3 days after ICV injection. The Nrf2 shRNA sequence was 5′-AAGCCTTACTCTCCCAGTGAATCGAAATTCACTGGGAGAGTAAGGCTT-3′ and a non-targeting RNA sequence serving as a negative control [35].

### Quantitative real-time PCR

Q-PCR was performed as previously described [36]. Total mRNA was isolated from tissues (Additional file 1: Figure S1) using the Trizol reagent (TAKARA, Japan) and measured using spectrophotometric analysis (OD260/OD280). After reverse transcription of total mRNA into cDNA, q-PCR analysis was performed with SYBR Green qPCR Master Mix. Nrf2 forward and reverse primers were 5′-CTACTCCCAGGTTGCCCACA-3′ and 5′-CGACTCATGGTCATCTACAAATGG-3′, respectively; HO-1 forward and reverse primers were 5′-GCTGGTGATGGCTTCCTTGTA-3′ and 5′-ACCTCGTGGAGACGCTTTACAT-3′, respectively; NQO-1 forward and reverse primers were 5′-ACGACAACGGTCCTTTCCAGA-3′ and 5′-CAGAAACGCAGGATGCCACT-3′, respectively; β-actin forward and reverse primers were 5′-GACAGGATGCAGAAGGAGATTACT-3′ and 5′TGATCCACATCTGCTGGAAGGT-3′, respectively. All samples were analyzed in triplicate with normalization to the β-actin in the Sham group. Relative quantification of mRNA expression was measured using the 2^−ΔΔCT^ method.

### Statistical analysis

The data were expressed as mean ± standard deviation (SD) and analyzed using an analysis of variance (ANOVA) followed by Tukey’s (one-way) or Bonferroni's (two-way) multiple comparisons post hoc test. Values of *P* < 0.05 were considered statistically significant. Analyses were performed using the SPSS version 20.0 software.

## Results

### Effects of Au on H_2_O_2_-induced primary cortical neurons

**Fig. 1:**
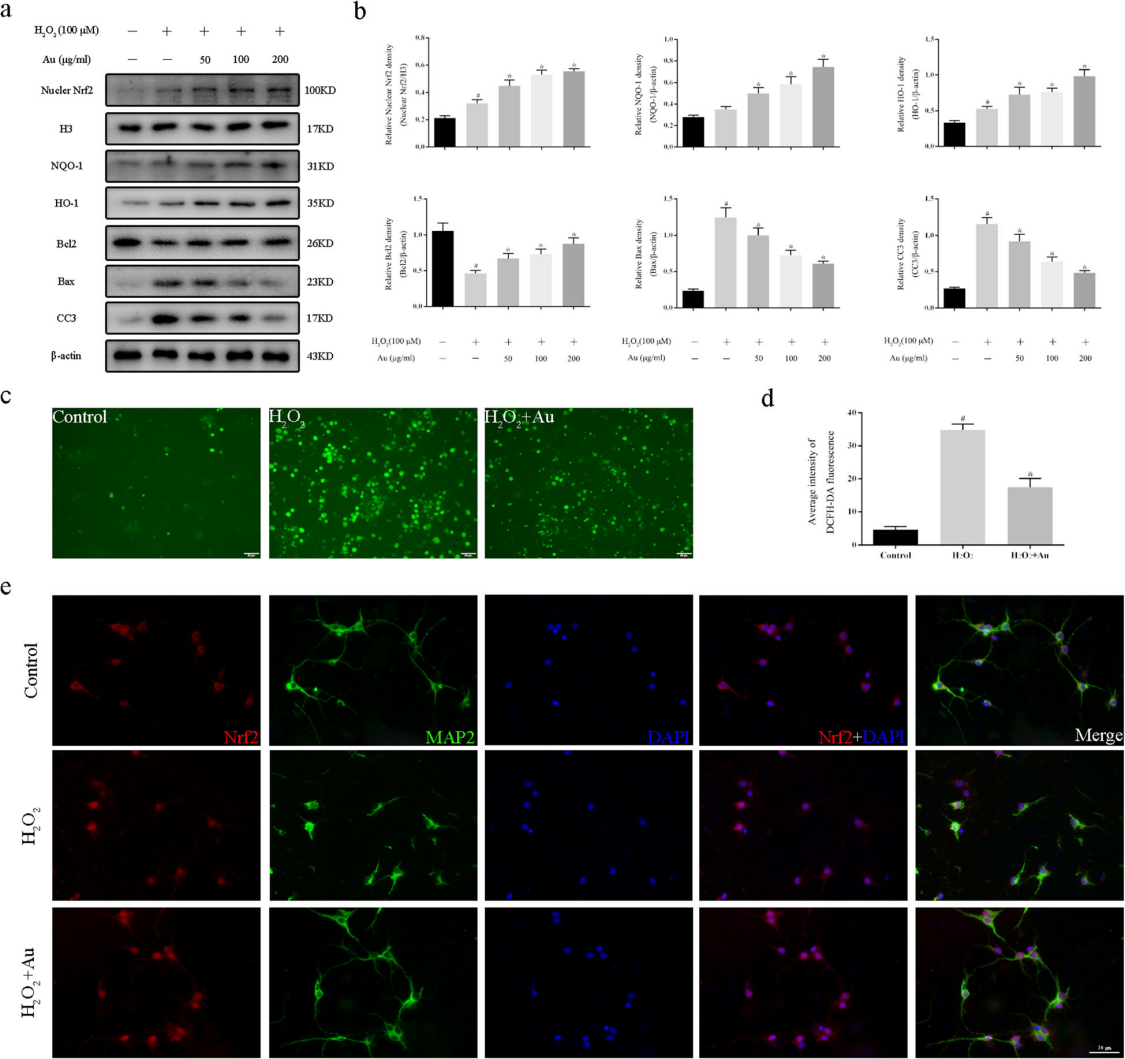
Effects of Au on the primary neurons exposed to 100 μM H_2_O_2_. **a**, **b** Representative western blot bands (**a**) and quantification of relative protein expression (**b**) for nuclear Nrf2, NQO-1, HO-1, Bcl2, Bax, and CC3. **c**, **d** Representative micrographs (**c**) and quantification (**d**) of DCFH-DA staining of Control group, H_2_O_2_ group, and H_2_O_2_ + Au (200 μg/ml) group in primary neurons. **e** Representative image of immunofluorescence staining of Control group, H_2_O_2_ group, and H_2_O_2_ + Au (200 μg/ml) group. Bars represent the mean ± SD. ^#^*P* < 0.05 versus Control group; *P < 0.05 versus H_2_O_2_ group. Scale bars = 50 μm (*n* = 3 for each group)

To determine the antioxidative effects of Au, we used H_2_O_2_ to stimulate primary neurons to construct a vitro model of oxidative stress. Western blot analysis showed that Au (50 μg/ml, 100 μg/ml, and 200 μg/ml) treatment significantly enhanced the cytoplasmic Nrf2 translocation to the nucleus and increased the expression of NQO-1, HO-1, and Bcl2 in a dose-dependent manner (Fig. 1a, b). In addition, western blot analysis also showed that Au reduced the expression of Bax and CC3 in neurons after H_2_O_2_ stimulation. As shown in Fig. 1 c and d, H_2_O_2_ significantly increased ROS levels when compared with those of the Control group, and this effect was reversed by Au (200 μg/ml) treatment. Immunofluorescence staining was consistent with the western blot results that Au promoted the cytoplasmic Nrf2 into the nucleus in conditions of oxidative damage (Fig. 1e).

### Au ameliorated brain edema and improved short-term neurologic functions after TBI

**Fig. 2:**
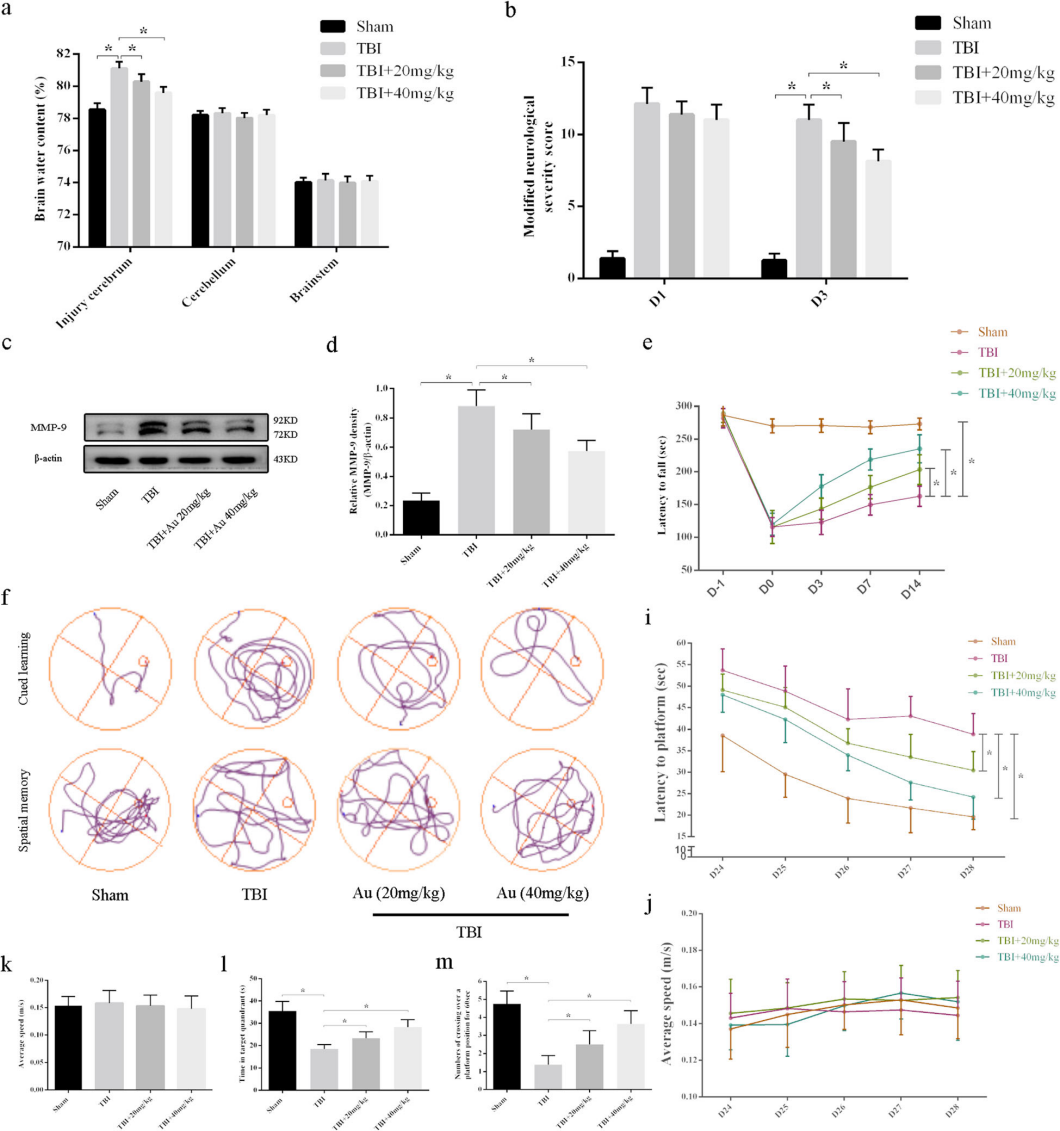
Au treatment reduced brain edema and improved neurologic functions. **a**, **b** Effects of TBI and Au on water content (**a**) and neurologic deficits (**b**). **c**, **d** Representative western blot bands (**c**) and quantification of relative protein expression (**d**) for MMP-9. **e** Effects of Au on the latency to fall in the rotarod test were analyzed using two-way ANOVA. The Bonferroni post hoc test was used to compare differences among several groups at 14 days post-TBI. **f** Typical swimming path of mice in all groups during the training days (upper) and the probe trial period (lower) of the MWM test. **i** Effects of Au on the latency to find platform in the MWM test. The two-way ANOVA was performed, and the Bonferroni post hoc test was utilized to compare differences among several groups on day 28. **j**, **k** Au did not affect the average swimming speed during the training days (**j**) or during the probe trial period (**k**). **l** Time spent in the correct quadrant during the probe trail. **m** Number of crossings over the platform position during the probe trail. Bars represent the mean ± SD. *P < 0.05 versus indicated groups

At 3 days after TBI, BWC increased significantly. Au (20 mg/kg or 40 mg/kg) treatment alleviated brain edema (Fig. 2a). As shown in Fig. 2c, d, TBI caused the increased expression of the MMP-9 protein, which destroys the blood-brain barrier. However, this effect was reversed by Au (20 mg/kg or 40 mg/kg) treatment. To further explore the neuroprotective effects of Au on TBI, we measured neurologic functions in the mice. Although Au therapy on the first day of trauma did not improve mNSS scores, it did improve significantly after 3 days of treatment (Fig. 2b). Furthermore, significantly greater recovery was observed in Au-treated TBI mice than in TBI mice as measured using the rotarod test (Fig. 2e).

### Au improved long-term neurologic functions after TBI

During the training days (Fig. 2j) or the probe trials (Fig. 2k), there was no difference in average swimming speed among the four groups. However, TBI significantly caused the mice to show prolonged escape latency in the cued learning phase. By contrast, mice in TBI + Au groups had a shortened escape latency compared with those in the TBI group, suggesting that Au improved the learning functions in TBI mice (Fig. 2i). In the probe trial period, the TBI mice treated with Au had significantly greater correct quadrant dwell time (Fig. 2l) and more platform crossings (Fig. 2f, m) than did TBI mice, suggesting that Au improved memory functions in TBI mice.

### Au promoted neurons survival after TBI

**Fig. 3:**
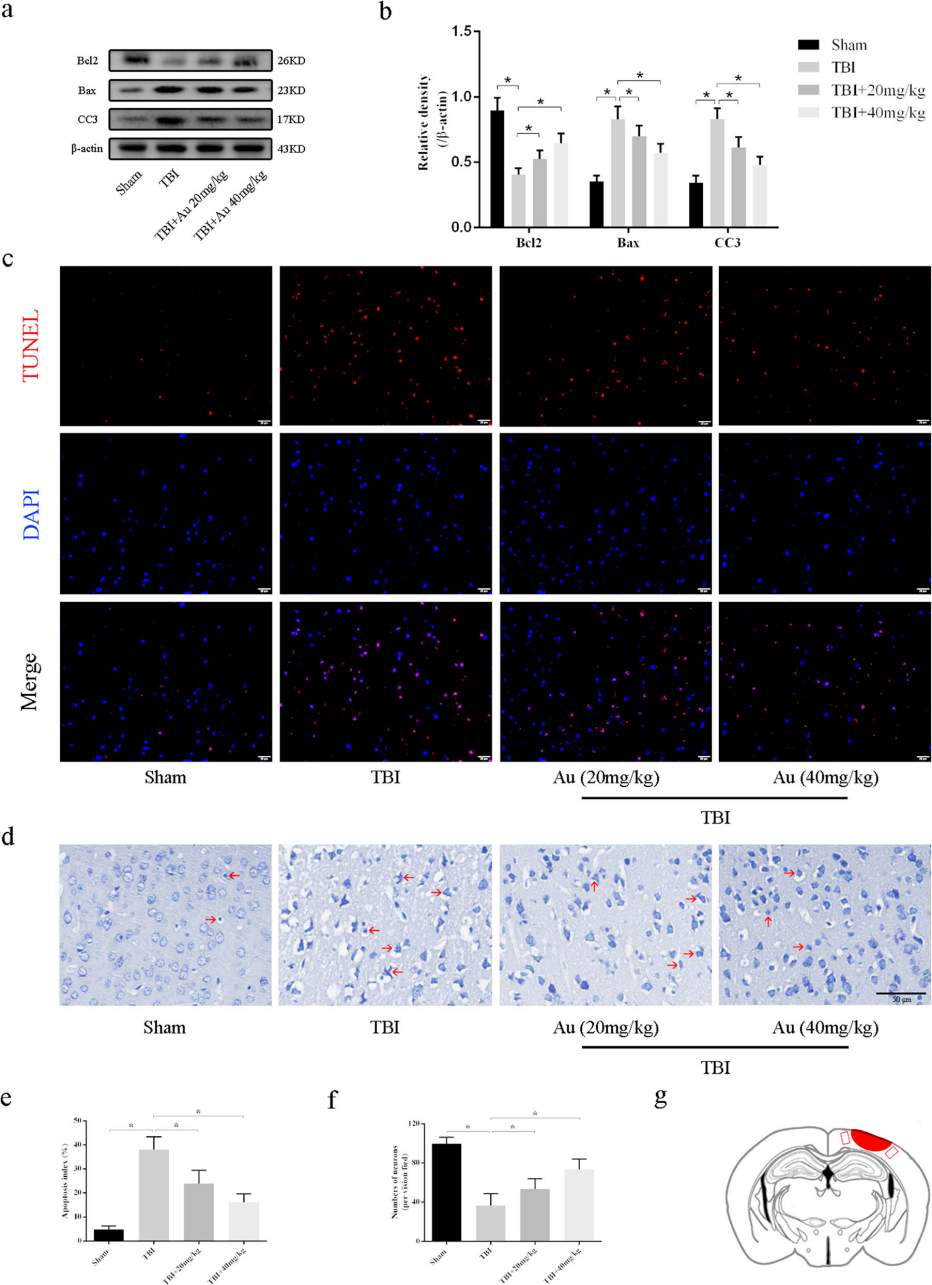
Au decreased the neural apoptosis and neuronal loss caused by TBI. **a**, **b** Representative WB bands (**a**) and quantification of relative protein expression (**b**) for Bcl2, Bax, and CC3. **c**–**f** Representative photomicrographs and quantification of TUNEL staining (**c**,** e** Scale bars = 20 μm) and Nissl staining (**d**, **f** Scale bars = 50 μm) in cortex of the traumatized side. The red arrow indicated damaged neurons. **g** Diagram of mouse brain section showing the location of lesion cavity (red area) and photograph region (red squares). Bars represent the mean ± SD. *P < 0.05 versus indicated groups (*n* = 6 for each group)

In the vivo studies, we also measured the expression of apoptosis-associated proteins, and western blot results were consistent with those in vitro experiments. Au (20 mg/kg or 40 mg/kg) treated-TBI mice had significantly elevated expression of Bcl2 and lower expression of Bax and CC3 than those of TBI mice (Fig. 3a, b). TUNEL staining showed that there were significantly more apoptotic positive cells in the TBI group than in TBI + Au groups (Fig. 3c, e). Then, we measured damaged neurons in the ipsilateral cortex surrounding the injury site. As shown in Fig. 3d, the neurons were clear and intact in the Sham group. By contrast, damaged neurons displayed irregular cell bodies, shrinkage, and hyperchromatic nuclei in significantly greater numbers in the TBI group. After Au administration, less neuron loss was found than in the TBI group (Fig. 3f). Meanwhile, we explored the effects of Au on neurons in the CA1 region of the hippocampus. TBI mice with Au (20 mg/kg or 40 mg/kg) administration had less apoptotic positive cells and neuronal loss (Additional file 1: Figure S3).

### Au attenuated oxidative stress after TBI

**Fig. 4:**
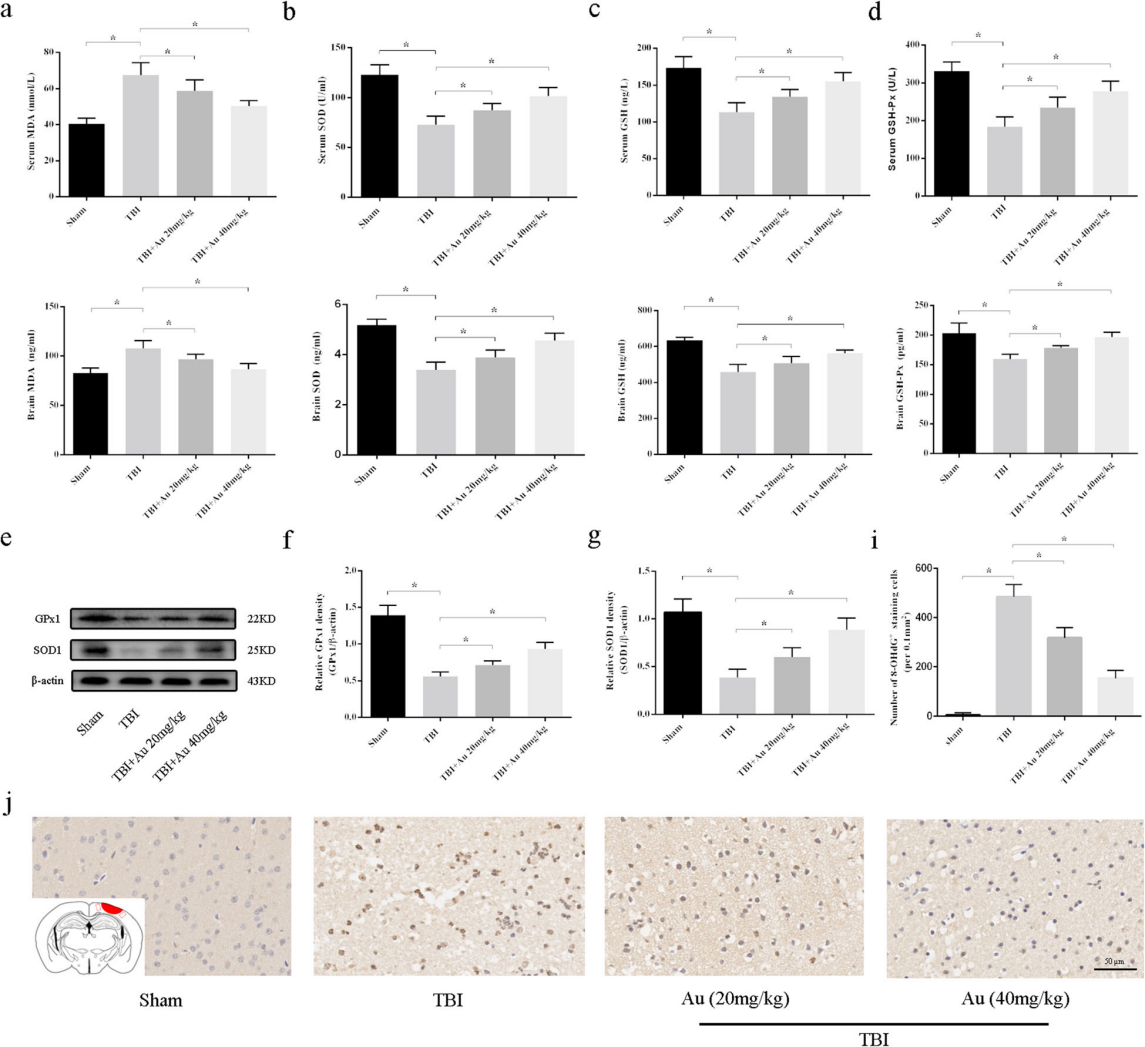
Au attenuates oxidative stress in TBI mice. **a**–**d** The ELISA analysis of MDA (**a**), SOD (**b**), GSH (**c**), and GSH-Px (**d**) in serum (upper) and brain tissue (lower) among four groups. **e**–**g** WB bands (**e**) and quantification of relative protein expression (**f**, **g**) for GPx1 and SOD1. **i**, **j** Representative immunohistochemical images and quantitative analysis of oxidative stress markers 8-OHdG. Bars represent the mean ± SD. *P < 0.05 versus indicated groups. Scale bars = 50 μm (*n* = 6 for each group)

SOD, MDA, GSH, and GSH-Px levels in serum and brain tissue were measured to determine whether Au suppressed TBI-induced oxidative damage. Both in serum and brain tissue, TBI significantly decreased SOD, GSH, and GSH-Px levels, whereas increased MDA levels. After Au application, mice in the TBI + Au groups (20 mg/kg or 40 mg/kg) displayed significantly higher serum levels and brain concentrations of SOD, GSH, and GSH-Px than did TBI mice. Furthermore, mice with Au injection had significantly lower concentrations of serum and brain MDA than did those in the TBI group (Fig. 4a–d). Similarly, western blot results showed that the protein expression of SOD1 and GPx1 significantly increased in the TBI + Au groups as compared with those of the TBI group (Fig. 4e–g). In Fig. 4i, j, IHC staining was performed to determine whether Au treatment inhibited the production of the oxidative stress marker 8-OHdG. We found that Au-treated mice had significantly fewer 8-OHdG ^+^cells than did TBI mice.

### Au promoted the expression and nuclear translocation of Nrf2 after TBI

**Fig. 5:**
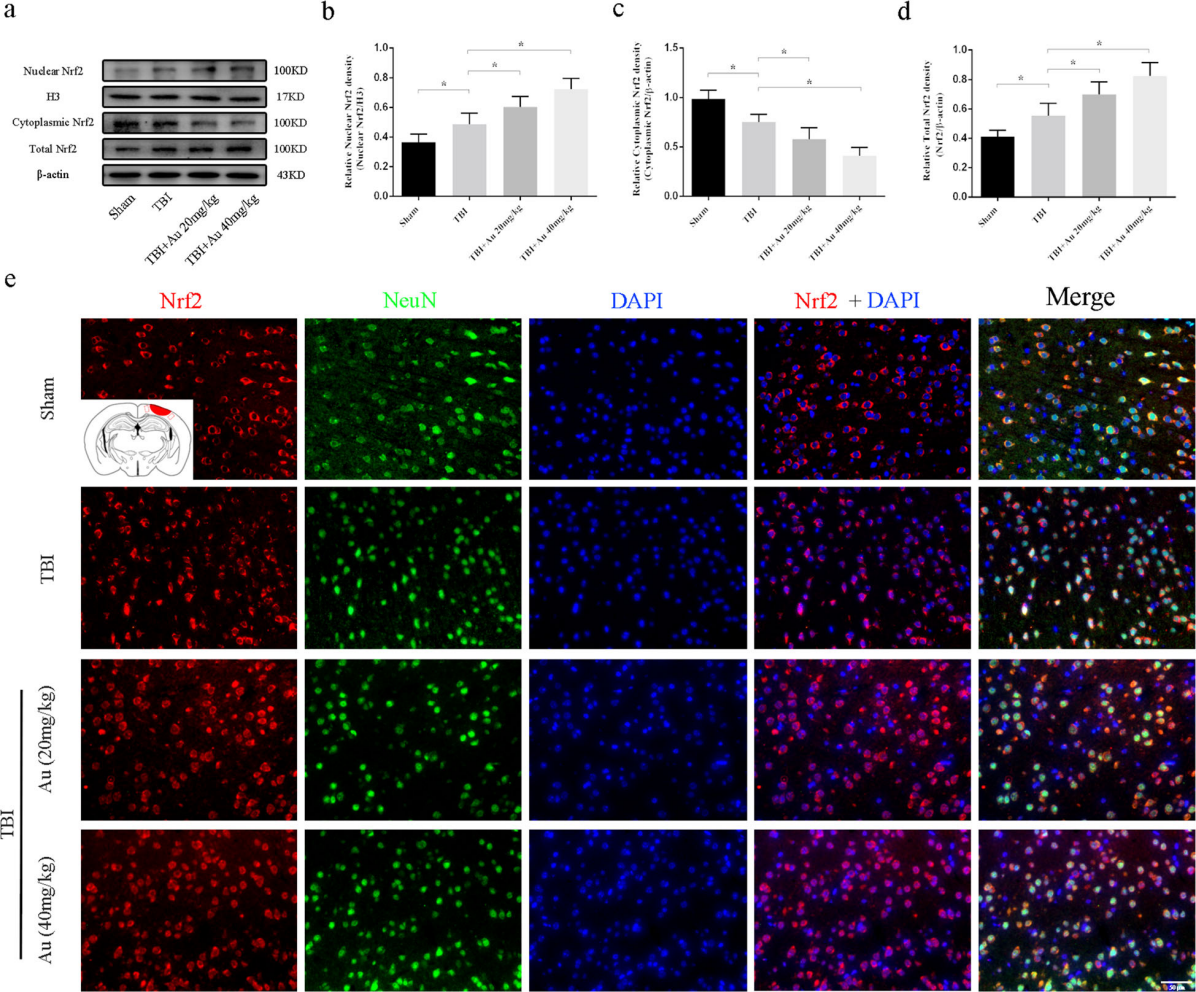
Effects of Au on protein level of Nrf2 in the perilesional cortex after TBI. **a**–**d** Representative WB bands (**a**) and quantification analysis of nuclear Nrf2 (**b**), cytoplasmic Nrf2 (**c**), and total Nrf2 (**d**). **e** Typical double immunofluorescence images of NeuN and Nrf2 (scale bars = 50 μm). Bars represent the mean ± SD. *P < 0.05 versus indicated groups (*n* = 6 for each group)

WB analyses suggested that TBI significantly increased total and nuclear Nrf2 expression compared with that of the Sham group. TBI mice with Au treatment had further increased expression of total and nuclear Nrf2 (Fig. 5a, b, d). Meanwhile, cytoplasmic Nrf2 protein levels in TBI + Au groups were significantly lower than those of the TBI group (Fig. 5a, c). Immunofluorescence staining confirmed our WB findings that Au treatment increased protein levels of total Nrf2 and enhanced Nrf2 nuclear translocation in neurons (Fig. 5e and Additional file 1: Figure S4).

### Au upregulated the expression of Nrf2 downstream proteins

**Fig. 6:**
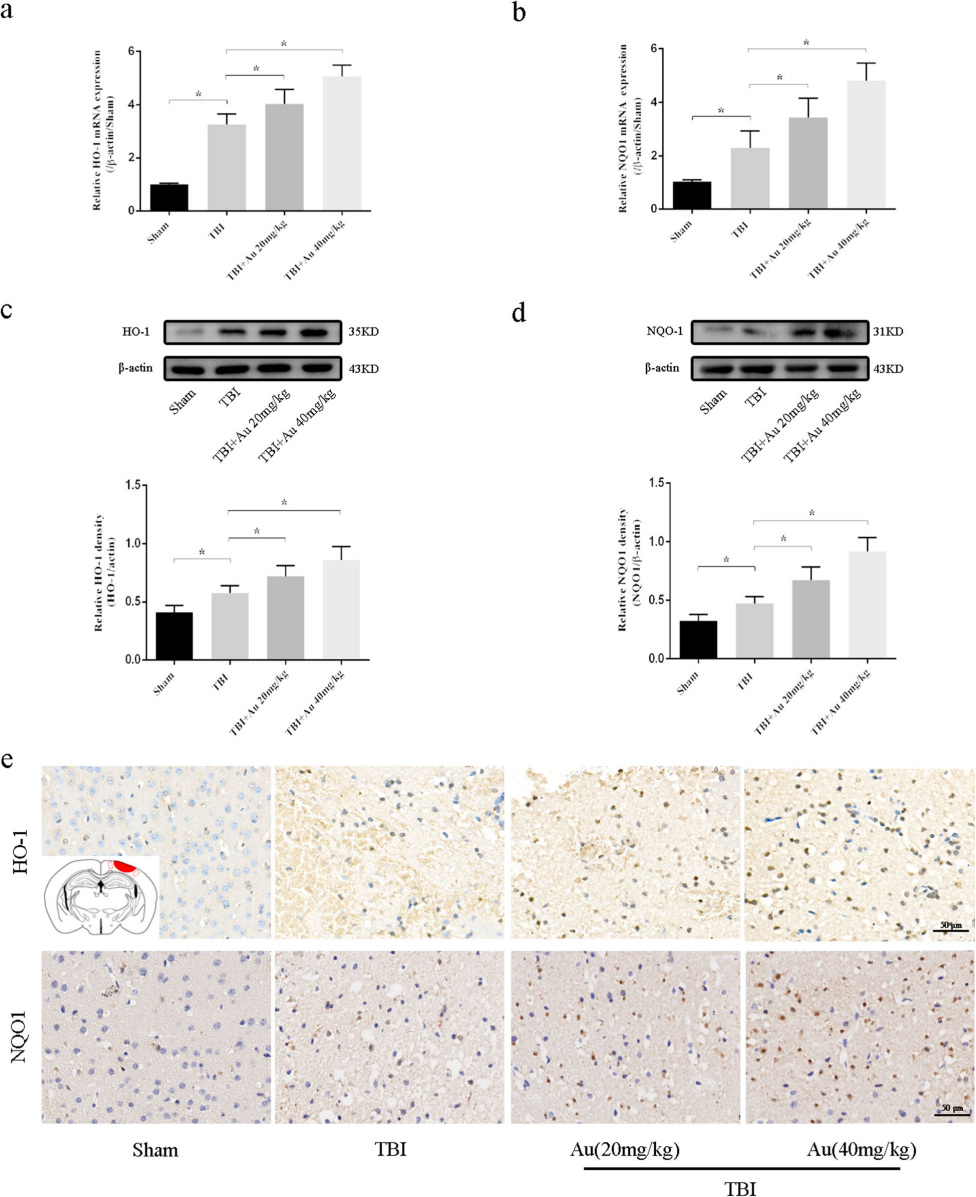
Au upregulated the expression of HO-1 and NQO-1. **a**, **b** The mRNA levels of HO-1 (**a**) and NQO-1 (**b**) in cerebral cortex of the injured side. **c**, **d** WB bands (top) and quantitative analysis (bottom) of HO-1 (**c**) and NQO1 (**d**). **e** Representative IHC images of HO-1 (upper) and NQO-1 (lower). Bars represent the mean ± SD. *P < 0.05 versus indicated groups. Scale bars = 50 μm

We measured downstream pathway expression of Nrf2 at the mRNA and protein levels. Q-PCR results showed that TBI increased both *HO-1* and *NQO-1* mRNA expression, and Au further distinctly enhanced their expression in a dose-dependent manner (Fig. 6a, b). Consistent with mRNA variations, WB results demonstrated that Au caused greater protein expression of HO-1 and NQO-1 than in the TBI group (Fig. 6c, d). These results indicated that Au induced the expression of HO-1 and NQO-1 at the transcriptional and translational levels. In addition, representative immunohistochemical images of HO-1 and NQO-1 proteins are shown in Fig. 6e.

### Au suppressed inflammatory response in the perilesional cortex after TBI

**Fig. 7:**
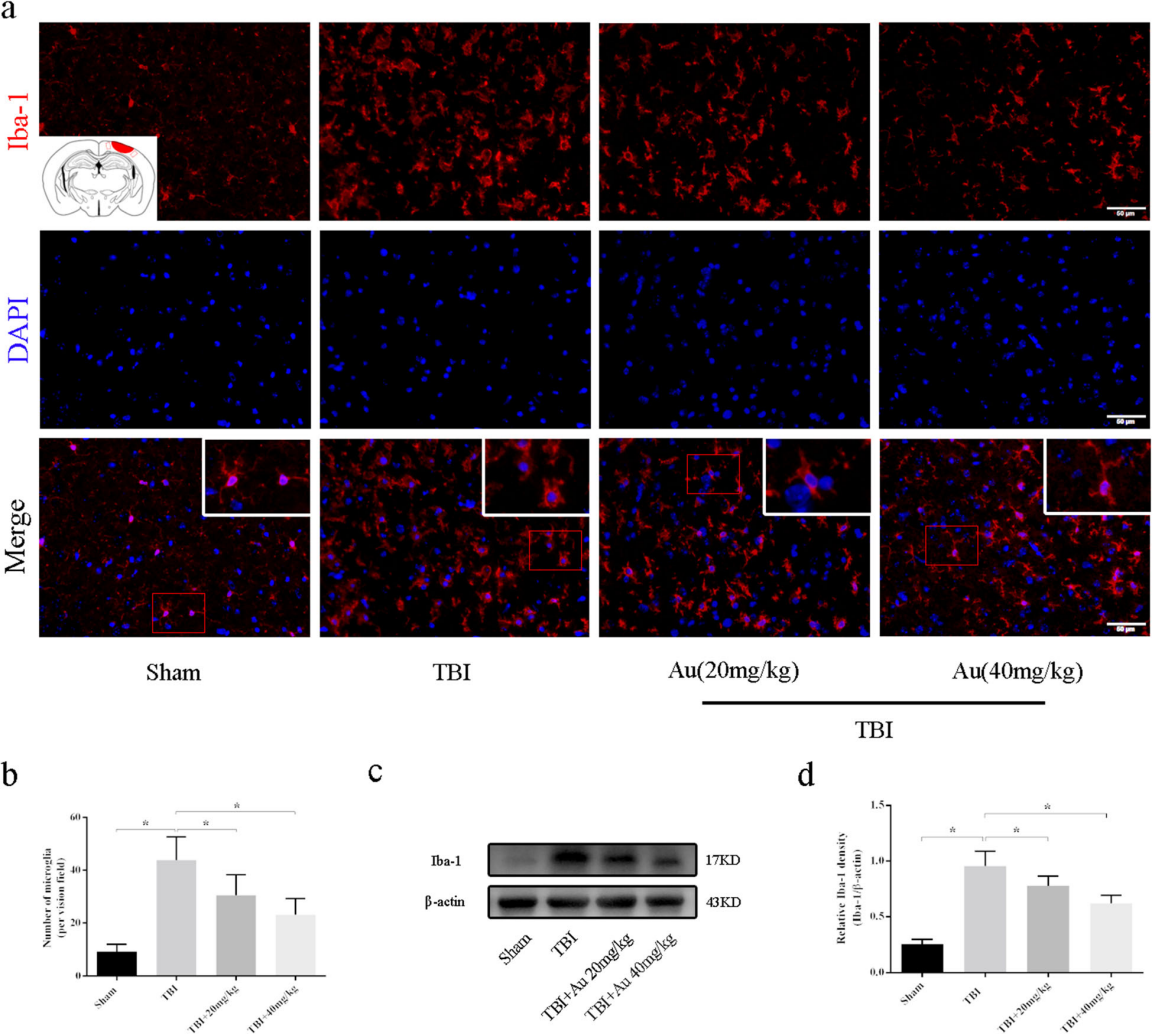
Effects of Au on the number of Iba-1+ cells in the perilesional cortex after TBI. **a**, **b** Representative photomicrographs (**a**) and quantitative analysis (**b**) of Iba-1-positive cells in the perilesional cortex. **c**, **d** Representative western blot bands (**b**) and quantification analysis of Iba-1 (**c**). Bars represent the mean ± SD. *P < 0.05 versus indicated groups. Scale bars = 50 μm (*n* = 6 for each group)

To measure microglial recruitment in the perilesional cortex, we used WB analyses and IF staining. As shown in Fig. 7 a and b, the number of microglia in the TBI + Au groups markedly reduced compared with that of TBI group. In addition, WB analysis showed that Au significantly weakened the increased expression levels of Iba-1 in the injured tissue (Fig. 7c, d). These results suggested that Au could inhibit microglial aggregation caused by TBI. Microglial activation is accompanied by morphological changes. Microglia from the TBI mice showed enlarged, round cell bodies, and shortened processes, while those in TBI + Au groups exhibited a less-activated phenotype with smaller cell bodies and elongated processes (Fig. 7a).

**Fig. 8:**
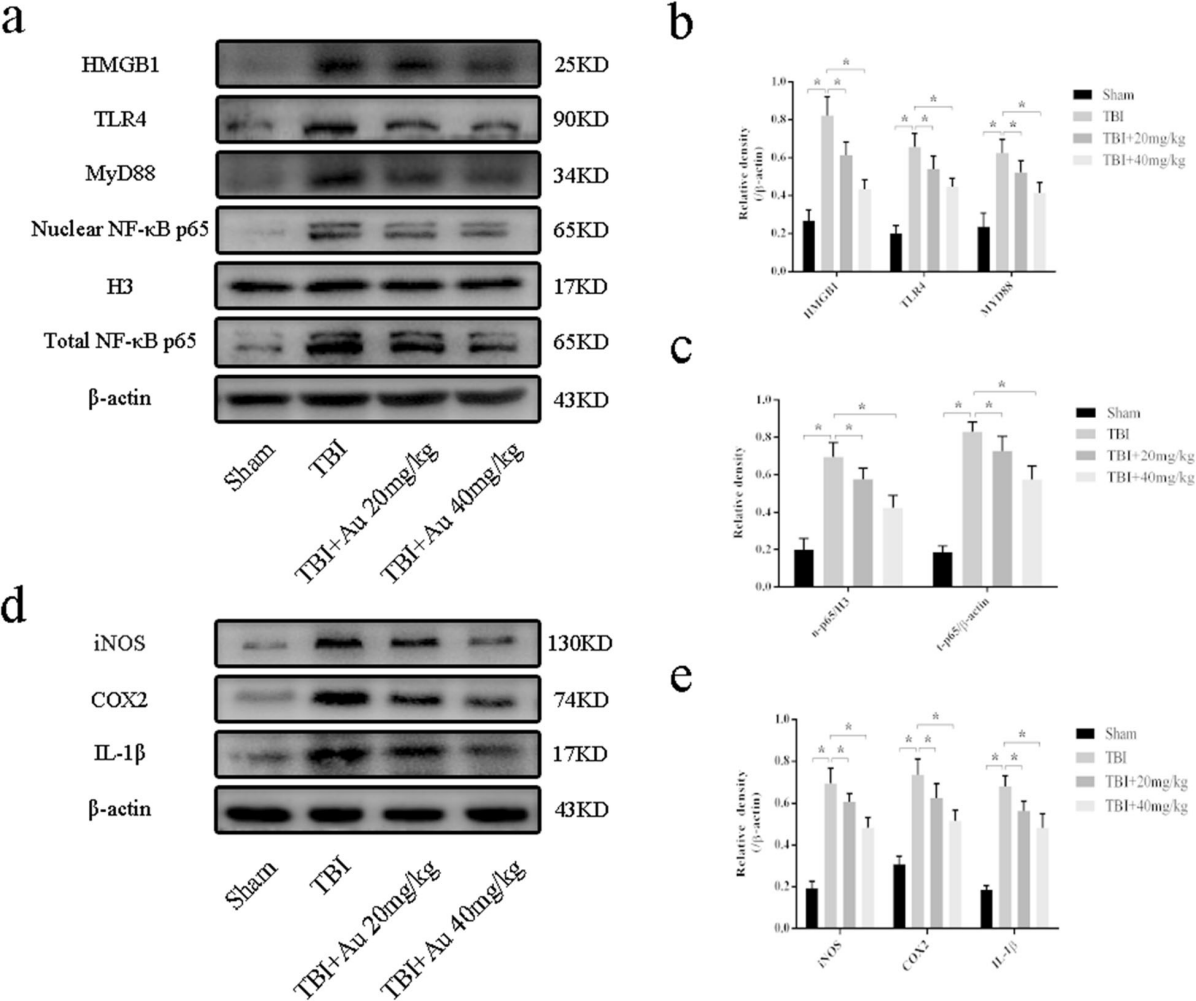
Au reduced HMGB1-meditated inflammation after TBI. **a**–**c** Representative western blot bands (**a**) and quantification analysis of HMGB1, TLR4, MyD88 (**b**), nuclear NF-κB p65, and total NF-κB p65 (**c**). **d**, **e** Representative western blot bands (**d**) and quantification analyses (**e**) of iNOS, COX2, and IL-1β. Bars represent the mean ± SD. *P < 0.05 versus indicated groups

Then, we detected the expression of HMGB1 and downstream proteins. Au-treated mice had lower levels of HMGB1, TLR4, MyD88, and total NF-κB p65 than did TBI mice (Fig. 8a–c). Furthermore, NF-κB p65 in the nucleus was reduced in the TBI + Au group (Fig. 8a, c). There were consistent changes in inflammatory cytokines (Fig. 8d, e).

### Nrf2 interference weakened the antioxidant and anti-inflammatory effects of Au on TBI

**Fig. 9:**
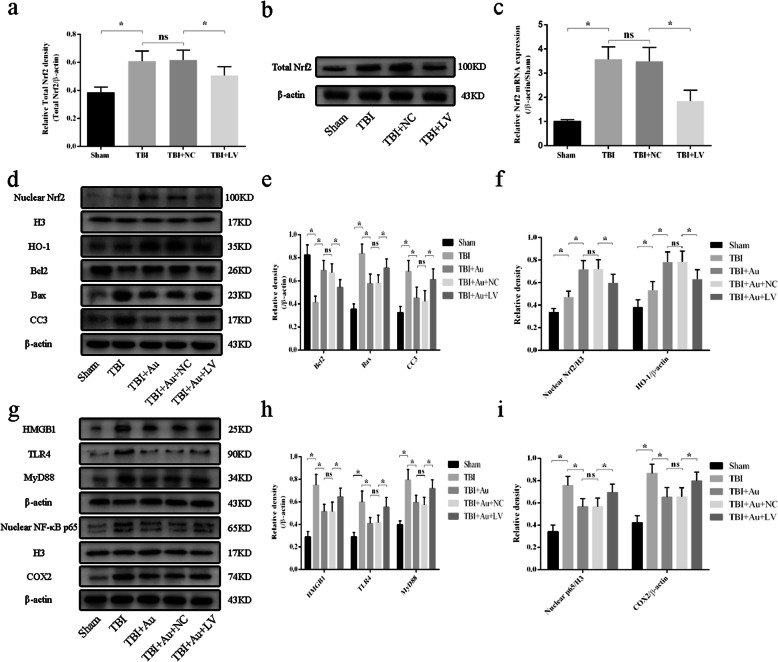
Effects of Nrf2 shRNA delivery and Nrf2 shRNA with Au co-administration in TBI mice. **a**, **b** WB bands (**a**) and quantification (**b**) of Nrf2 protein in Sham, TBI, TBI + NC, and TBI + LV groups. **c** The mRNA expression levels of Nrf2 in all groups. **d**–**i** Representative western blot bands (**d**, **g**) and quantification analysis of nuclear Nrf2, HO-1, Bcl2, Bax, CC3 (**e**, **f**), HMGB1, TLR4, MyD88, nuclear NF-κB p65, and COX2 (**h**, **i**). Bars represent the mean ± SD. *P < 0.05 versus indicated groups; ns, not significant (*n* = 6, each group)

We wondered whether the neuroprotective effect of Au depended on Nrf2 activation after TBI. We applied Au (40 mg/kg) together with Nrf2 shRNA co-treatment. To test the effect of Nrf2 interference, q-PCR and WB analyses were performed. The results showed that Nrf2 shRNA significantly depressed expression of Nrf2 at the mRNA and protein levels (Fig. 9a–c). We further evaluated protein levels of nuclear Nrf2. Au and Nrf2 shRNA co-treated mice displayed a reduction in nuclear Nrf2 compared with that of the TBI + Au + NC group (Fig. 9d, f). Expression levels of HO-1 and Bcl2 were notably decreased in TBI + Au + LV groups, whereas Au-treated mice with Nrf2 interference showed increased protein expression of Bax and CC3 (Fig. 9d–f).

Finally, we measured expression of HMGB1-TLR4 pathway components. The results indicated that Nrf2 knockdown significantly increased expression levels of HMGB1, TLR4, MyD88, nuclear NF-κB p65, and COX2 as compared with those of the TBI + Au + NC group (Fig. 9g–i).

## Discussion

Secondary brain injury has complex and varied pathophysiological mechanisms. Substantial evidence suggests that oxidative stress participates in secondary brain injury [4, 10, 11, 17]. Recently, studies have demonstrated that Au has potent antioxidant effects and reduces excessive production of ROS in non-alcoholic fatty liver disease and acute lung injury [37, 38]. Until now, few experiments investigated the potential antioxidative in CNS diseases, except that Xue and colleagues reported the antioxidant bioactivities of Au in P12 cells (a neuronal cell line) and in a rat model of diabetic encephalopathy [27–29]. In the present study, we first investigated the antioxidative effects of Au on primary neurons. We found that Au inhibited intercellular ROS generation, restored the balance between the expression of Bcl2 and Bax, and suppressed caspase-3 activation. These results are consistent with those of previous studies [27, 37]. Then, we further tested the effectiveness of Au in TBI mice. We found that Au administration substantially reduced MDA content and numbers of 8-OHdG-positive cells, which are markers of oxidative stress and indicate the presence of cellular damage and destruction [39]. Concomitant with the decreased generation of ROS, Au also increased brain and serum levels of essential antioxidant enzymes that inhibit the overproduction of ROS. These findings suggest that Au activates ARE to regulate cellular oxidant–antioxidant balance. TBI causes cerebral cortex damage that has been confirmed to affect a series of neurobehavioral functions [40]. In addition to motor deficits, TBI mice in our experiments exhibited cognitive impairments that were mitigated by Au treatment. This suggests that Au augments brain tissue repair and promotes functional recovery. Published literatures have shown that the neuroprotective effect of Au was both in the short term and the long term. The mechanisms involve in oxidative stress-induced neuronal loss [41]. After Au treatment, attenuation of oxidative stress could reduce neuronal loss in hippocampus [27–29]. Similarly, we found that Au decreased the loss of neurons both in cortex and hippocampus in a mouse TBI model. We hypothesized that the improvement of neurologic functions in TBI mice was probably due to the fact that Au alleviated oxidative stress.

Nrf2 plays an indispensable role in maintaining cellular homeostasis. Substantial lines of evidence have suggested that it is beneficial for CNS diseases, including TBI [13–15]. Under physiologic conditions, Nrf2 binds with Keap1 in the cytoplasm. However, under conditions of injury, Nrf2 dissociates from Keap1 and translocates into the nucleus, where it activates the Nrf2-ARE pathway. Au has been demonstrated to have the property to promote Nrf2 into nuclear, which triggers Nrf2-induced antioxidative signaling pathway [31, 32]. In both in vitro and in vivo studies, we did find that Au enhanced nuclear accumulation of Nrf2 and increased cytoplasmic expression of HO-1 and NQO1 in neurons. While Nrf2 was downregulated, the antiapoptotic and antioxidative properties were abrogated. These results indicate that Au ameliorated TBI in an Nrf2-dependent manner.

Inflammation is one of the major determinants of secondary brain damage after TBI [5]. Activated microglia, the principle resident macrophages of the CNS, produce and release a number of pro-inflammatory cytokines and chemokines. These cytokines activate and recruit more inflammatory cells to amplify the inflammatory response [42]. Au restrains the activation of astrocytes and microglia [26, 30]. Our results showed that Au inhibited the activation of microglia and prevented them from accumulating in the injury cortex. Moreover, we observed that Au markedly reduced protein levels of inflammatory cytokines. These findings suggest that Au also provides neuroprotective effects in a TBI model via anti-inflammation, whereas the anti-inflammatory role of Au was abrogated by Nrf2 knockdown. Consistent with these results, a recent study has shown that Nrf2 knockdown in RAW267.4 cells diminished the bioactivity of Au to impede inflammation, indicating that Au-inhibited inflammation dependents on Nrf2 expression [32].

In TBI, there is crosstalk between oxidative and inflammation. ROS deplete cellular antioxidants, react with nucleotides and proteins, and induce peroxidation of cellular membranes, further damaging cells and organelles, which initiates or intensifies inflammation [6, 8]. Damaged neurons and activated microglia passively and actively release HMGB1 into the extracellular milieu, respectively [20, 21]. HMGB1 binds with TLR4, a member of the TLR family, initiating the MyD88-dependent pathway leading to direct NF-κB activation and inducing the production of pro-inflammatory genes and chemokines [22–24]. A study has reported reduced expression of HMGB1 caused by Au treatment in epileptic mice [30]; however, the mechanism remains unclear. In the TBI model, our studies showed that Au inhibited HMGB1-TLR4 signaling pathway-mediated neuroinflammation, but this process relies on the expression of Nrf2. We hypothesize that attenuation of oxidative stress reduces the release of HMGB1, which alleviates the inflammatory response. Similarly, Zeng and Wang have reported that elevated antioxidant response reduces the inflammation and weakens the brain damage in the TBI model [10, 12].

There are several potential limitations deserving attention in our experiments. First, although studies have shown that Au plays a protective role by phosphorylating AMPK to regulate Nrf2 translocation into the nucleus [31, 32], we did not explore the specific molecular mechanism in TBI mice. Second, we only used Nrf2 shRNA to explore the mechanism, which might not have had an efficient inhibitor effect. Therefore, the data from Nrf2 knockout mice might be more persuasive.

## Conclusions

In summary, we provide evidence that Au has antioxidative and anti-inflammatory bioactivities in a TBI model. The underlying molecular mechanisms of these beneficial effects involve in regulation of ROS and HMGB1-mediated inflammation by trigger Nrf2-ARE signaling pathway. Our experimental results might provide a novel therapeutic strategy for TBI.

## Supplementary information

**Additional file 1: **Supplemental Figure 1: Representative image of the cortical lesion caused by a weight-drop system. Shaded areas illustrate the perilesional cortex that was harvested for WB, ELISA and q-PCR analysis. Supplemental Figure 2: Modified Neurological Severity Score points. 1 score is awarded for the inability to perform the test or for the lack of a tested reflex (normal score, 0; maximal score, 18). Supplemental Figure 3: Au alleviated the neural apoptosis and neuronal loss in hippocampus. (a, c) Representative photomicrographs and quantification of Nissl staining in the CA1 region of hippocampus (scale bars = 20 μm). (b, d) Representative photomicrographs and quantification of TUNEL staining in the CA1 region of hippocampus. Red, TUNEL; blue, DAPI (scale bars = 50 μm). (e) Diagram of mouse brain section showing the location of lesion cavity (red) and photograph region (red square). Bars represent the mean ± SD. (n=6 for each group, one-way ANOVA, *P < 0.05 versus indicated groups). Supplemental Figure 4: Quantification of Nrf2 fluorescent intensity (one-way ANOVA, *P < 0.05 versus indicated groups).

## Data Availability

All data during this study are included in this article.

## Abbreviations

Au: Aucubin
TBI: Traumatic brain injury
TUNEL: TdT-mediated dUTP Nick-End Labeling
q-PCR: Quantitative real time polymerase chain reaction
ELISA: Enzyme linked immunosorbent assay
Nrf2: Nuclear factor erythroid-2 related factor 2
ROS: Reactive oxygen species
HMGB1: High mobility group box 1
DAMPs: Damage associated molecular patterns
CNS: Central nervous system
Keap1: Kelch-like ECH-associated protein 1
HO-1: Heme oxygenase-1
NQO-1: NAD(P)H: quinone oxidoreductase-1
GPx: Glutathione peroxidase
GST: Glutathione-S-transferase
SOD: Superoxide dismutase
TLRs: Toll-like receptors
NF-κB: Nuclear factor-κB
MyD88: Myeloid differentiation factor 88
mNSS: Modified Neurological Severity Scores
MWM: Morris Water Maze
BWC: Brain water content
WB: Western blot
IF: Immunofluorescence
IHC: Immunohistochemistry
NC: Negative control
LV: Lentiviral vectors
Bcl2: B-cell lymphoma-2
Bax: Bcl-2 Associated X Protein
CC3: Cleaved-caspase 3
COX2: Cyclooxygenase-2
iNOS: Inducible Nitric Oxide Synthase
IL-1β: Interleukin-1β
ECL: Enhanced chemiluminescence
DCFH-DA: M29,79-dichlorodihydrofluorescein diacetate
8-OHdG: 8-Hydroxyguanosine
ICV: Intracerebroventricular
