# Supplementary material for: Aucubin alleviates oxidative stress and inflammation via Nrf2-mediated signaling activity in experimental traumatic brain injury
Source: J Neuroinflammation. 2020 Jun 15;17:188. doi: 10.1186/s12974-020-01863-9 (PMC7294631; doi:10.1186/s12974-020-01863-9)
Supplement: Supplementary file 1 — Additional file 1: Supplemental Figure 1: Representative image of the cortical lesion caused by a weight-drop system. Shaded areas illustrate the perilesional cortex that was harvested for WB, ELISA and q-PCR analysis. Supplemental Figure 2: Modified Neurological Severity Score points. 1 score is awarded for the inability to perform the test or for the lack of a tested reflex (normal score, 0; maximal score, 18). Supplemental Figure 3: Au alleviated the neural apoptosis and neuronal loss in hippocampus. (a, c) Representative photomicrographs and quantification of Nissl staining in the CA1 region of hippocampus (scale bars = 20 μm). (b, d) Representative photomicrographs and quantification of TUNEL staining in the CA1 region of hippocampus. Red, TUNEL; blue, DAPI (scale bars = 50 μm). (e) Diagram of mouse brain section showing the location of lesion cavity (red) and photograph region (red square). Bars represent the mean ± SD. (n=6 for each group, one-way ANOVA, *P < 0.05 versus indicated groups). Supplemental Figure 4: Quantification of Nrf2 fluorescent intensity (one-way ANOVA, *P < 0.05 versus indicated groups). [file 12974_2020_1863_MOESM1_ESM.docx]

ADDITIONAL FILES

**Aucubin alleviates oxidative stress and inflammation via Nrf2-mediated signaling activity in experimental traumatic brain injury**

Han Wang^1, 2†^, Xiao-Ming Zhou^3†^, Ling-Yun Wu^1†^, Guang-Jie Liu^1^, WeiDong Xu^2^, Xiang-Sheng Zhang^1^, Yong-Yue Gao^1^, Tao Tao^1^, Yan Zhou^1^, Yue Lu^1^, Juan Wang^1^, ChuLei Deng^2^, Zong Zhuang^1^, ChunHua Hang^1*^ and Wei Li^1*^

1. Department of Neurosurgery, Nanjing Drum Tower Hospital, The Affiliated Hospital of Nanjing University Medical School, Nanjing, China
2. Department of Neurosurgery, Jinling Hospital, the first School of Clinical Medicine, Southern Medical University, Nanjing, China
3. Department of Neurosurgery, Jinling Hospital, School of Medicine, Nanjing University, Nanjing, China

†Han Wang, Xiao-Ming Zhou and Ling-Yun Wu contributed equally to this work.

^*^**Corresponding author:**  ­­

Wei Li, lwxzlw@126.com, Tel./fax: +86 25 83106666-11901;

Chun-Hua Hang, hang_neurosurgery@163.com, Tel./fax: +86 25 83106666-11902.


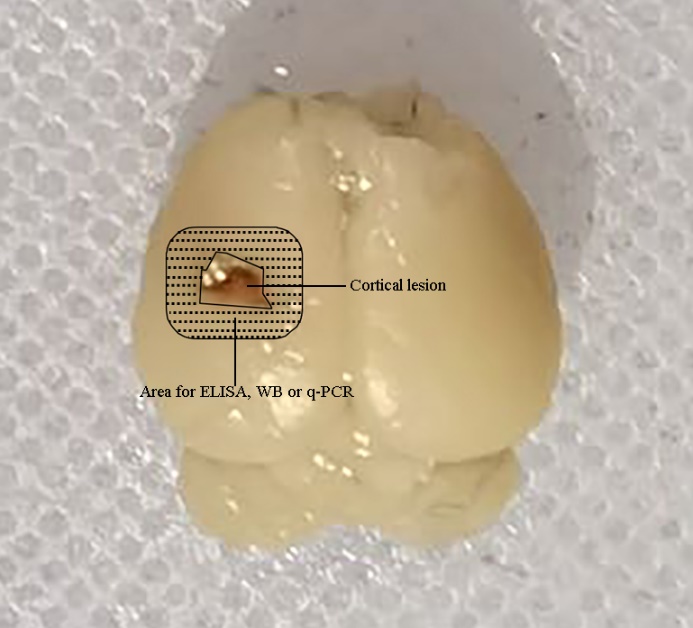


**Supplemental Figure 1:** Representative image of the cortical lesion caused by a weight-drop system. Shaded areas illustrate the perilesional cortex that was harvested for WB, ELISA and q-PCR analysis.


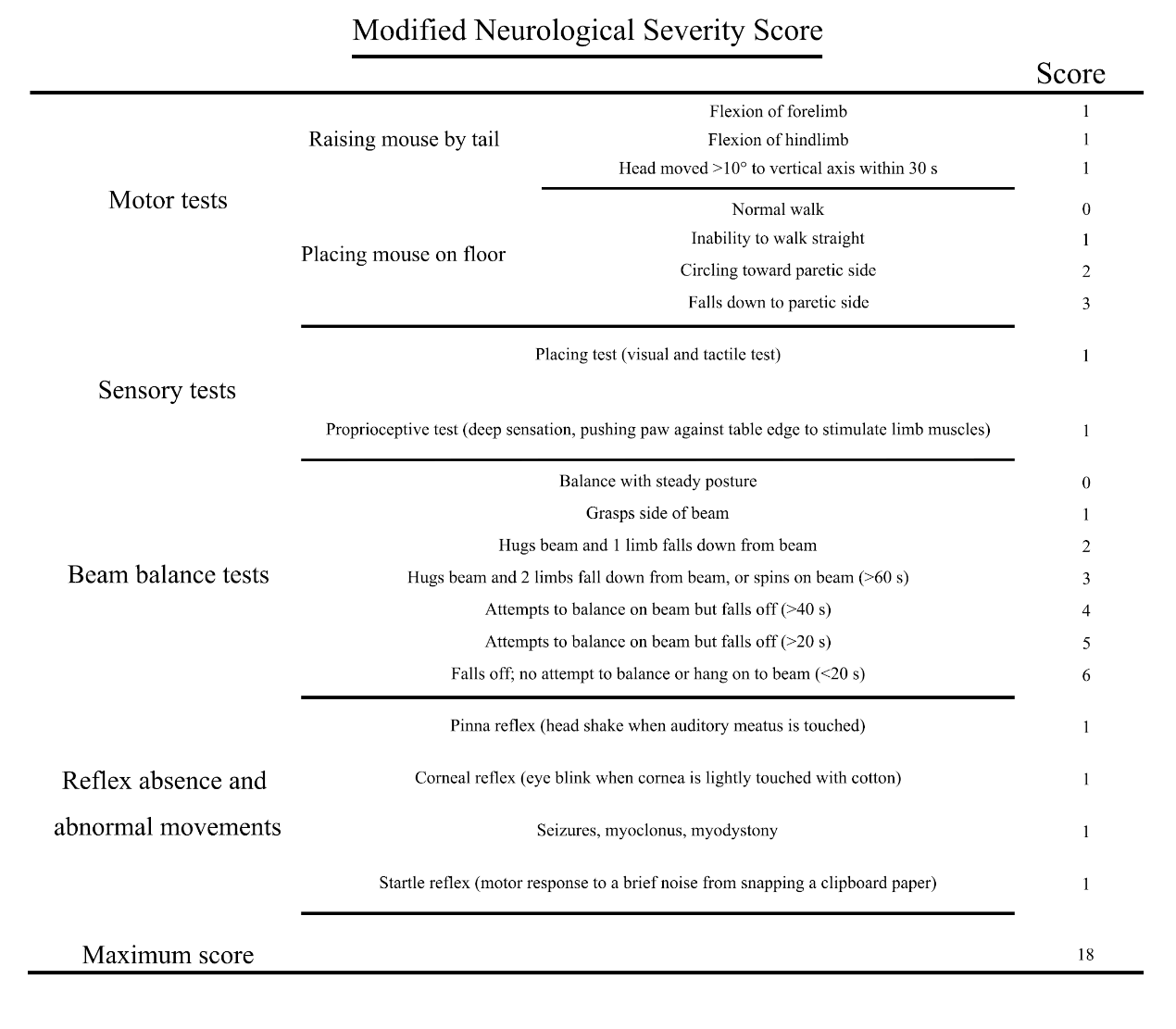


**Supplemental Figure 2:** Modified Neurological Severity Score points. 1 score is awarded for the inability to perform the test or for the lack of a tested reflex (normal score, 0; maximal score, 18).


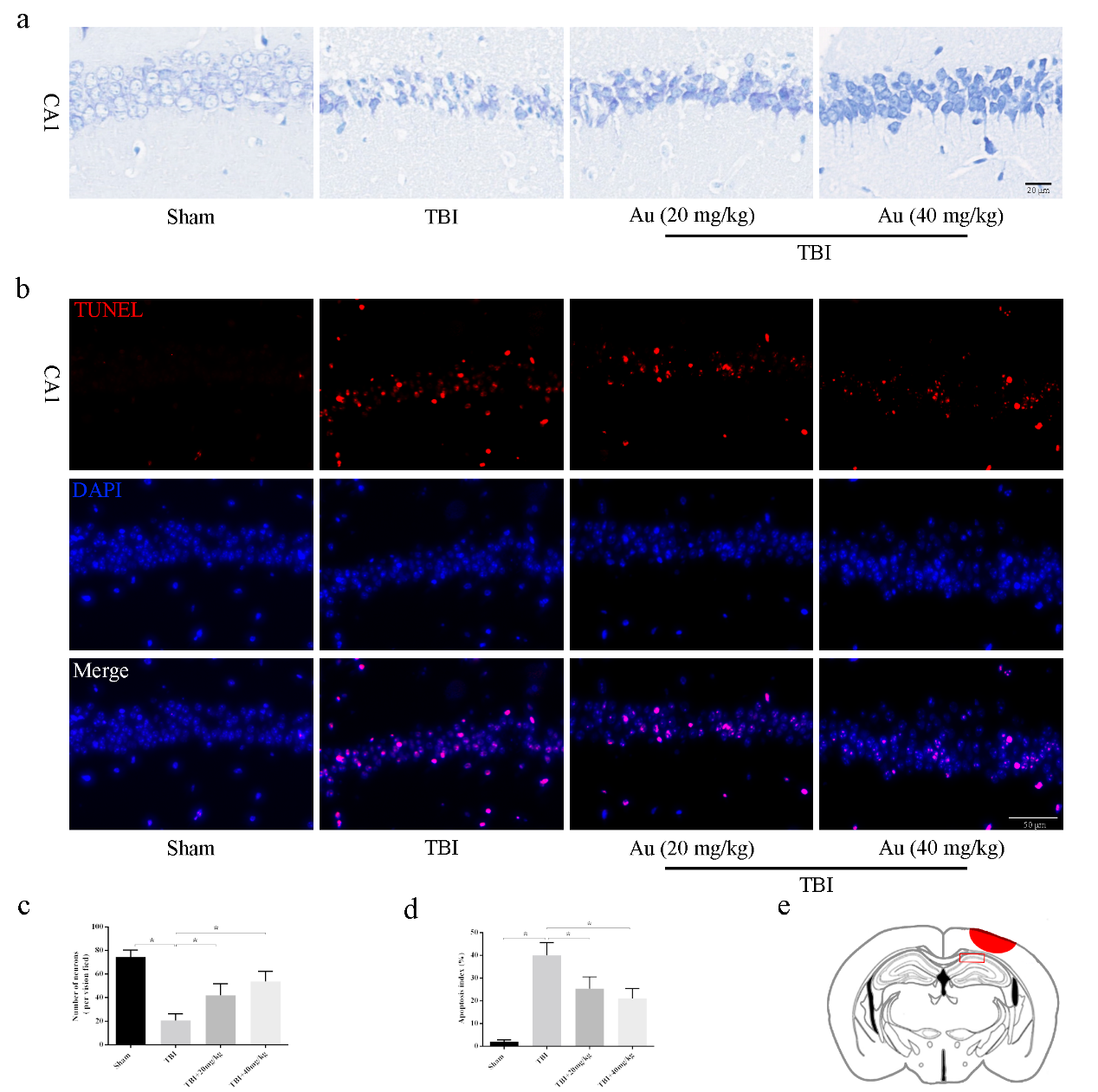


**Supplemental Figure 3:** Au alleviated the neural apoptosis and neuronal loss in hippocampus. (a, c) Representative photomicrographs and quantification of Nissl staining in the CA1 region of hippocampus (scale bars = 20 μm). (b, d) Representative photomicrographs and quantification of TUNEL staining in the CA1 region of hippocampus. Red, TUNEL; blue, DAPI (scale bars = 50 μm). (e) Diagram of mouse brain section showing the location of lesion cavity (red) and photograph region (red square). Bars represent the mean ± SD. (n=6 for each group, one-way ANOVA, **P* < 0.05 versus indicated groups)





**Supplemental Figure 4:** Quantification of Nrf2 fluorescent intensity (one-way ANOVA, **P* < 0.05 versus indicated groups).
